# Local Relapse and Survival Outcomes in Patients with Scalp Sarcoma: A Retrospective Study of 95 Patients Treated in a Provincial Cancer Care Institution Over 25 Years

**DOI:** 10.7759/cureus.5236

**Published:** 2019-07-25

**Authors:** Katie D Jasper, Caroline L Holloway, Kimberly J DeVries, Pauline T Truong

**Affiliations:** 1 Radiation Oncology, British Columbia Cancer Agency, Vancouver Centre, University of British Columbia, Vancouver, CAN; 2 Radiation Oncology, British Columbia Cancer Agency, Victoria Centre, University of British Columbia, Victoria, CAN; 3 Population Oncology, Cancer Surveillance & Outcomes, British Columbia Cancer Agency, Vancouver Centre, Vancouver, CAN; 4 Radiation Oncology, British Columbia Cancer Agency – Vancouver Island Centre, Victoria, CAN

**Keywords:** scalp, sarcoma, local relapse, overall survival, adjuvant radiotherapy

## Abstract

Objectives

There is limited literature on the optimal treatment of sarcoma arising in the scalp. This study evaluates local relapse (LR) and survival outcomes of patients with scalp sarcoma treated at a provincial cancer care institution.

Methods

A retrospective review of 95 patients with a primary diagnosis of scalp sarcoma referred from 1990-2015 was completed. Kaplan-Meier statistics were used to estimate LR-free survival (LRFS) and overall survival (OS). Survival curves were compared using log-rank tests. Regression analyses were performed using Cox proportional hazards model.

Results

The median age at diagnosis was 77 years. The most common histologies were angiosarcoma (27%), undifferentiated pleomorphic sarcoma (24%), and pleomorphic dermal sarcoma (21%). Final margins were 36% positive, 28% close, 31% negative, and 5% unknown. Of 73 patients treated with curative-intent, 32 (44%) experienced LR. Five-year LRFS was 56.0% and overall survival was 48.3%. Patients with close or positive margins who received pre- or post-operative radiotherapy (n=19) had similar LR risk compared to patients who did not (n=34) (five-year LRFS 41.8% vs 69.1%; p=0.145). On multivariate analysis, angiosarcoma was associated with a higher LR risk (Hazard ratio (HR) 12.06, p<0.001). The use of radiotherapy showed a trend towards reduced LR risk but did not reach statistical significance (HR 0.37, p=0.066).

Conclusions

Patients with scalp sarcoma have high risk of LR, particularly in cases with positive margins. Adjuvant radiation was not associated with improved local control for close or positive margins. Complete surgical excision to establish negative margins remains the primary standard treatment for patients with this rare disease.

## Introduction

Soft tissue sarcomas (STS) arising from the scalp are a rare, heterogeneous group of tumors that can be challenging to treat. An estimated 12,750 new cases of STS, or 3.9 cases per 100,000, will be diagnosed in the United States in 2019 [1]. The annual incidence of STS in Canada is 3.6 cases per 100,000 [2]. The most common sites include the extremities, viscera, and retroperitoneum [3]. Only 5% of STS arise in the head and neck region [4]. Due to anatomical constraints that limit the ability to obtain wide surgical margins, high rates of local relapse (LR) with poor overall prognoses have historically been seen in all head and neck STS [5-6]. Given the rarity of scalp STS there is limited literature to guide optimal treatment, and treatment is often guided by multidisciplinary expert opinions. Radical surgical excision with pre- or post-operative radiotherapy (RT) remains the standard treatment based on outcomes from all STS sites [7-8]. We undertook a retrospective study of patients diagnosed with scalp STS in British Columbia (BC), Canada to evaluate the rates of LR, distant recurrence (DR), and overall survival (OS) and to explore the effect of adjuvant RT on local control (LC) for patients with close or positive surgical margins. We hypothesized that patients with scalp STS would have poor LR-free survival (LRFS) and OS and adjuvant RT for close or positive margins would improve LRFS.

## Materials and methods

Our study included all BC Cancer patients referred with a primary diagnosis of STS located on the scalp between 1990 and 2015 (n=109). After anonymization, each case was reviewed to confirm proper diagnosis and location. The scalp was defined as the hair-bearing portion of the head. During the study period, the World Health Organization (WHO) classification of soft tissue tumors was updated [9]. We included all malignant and intermediate soft tissue histologies as defined by WHO [9]. The intermediate histologies within our population included solitary fibrous tumor, dermatofibrosarcoma protuberans (DFSP), and atypical fibroxanthoma (AF). Cases classified as malignant fibrous histiocytoma were included in the pleomorphic dermal sarcoma (PDS) group and cases of spindle cell sarcomas were reviewed to determine their origin. If dermal, they were included in the PDS group. If deep, they were included in the undifferentiated pleomorphic sarcoma (UPS) group. Excluded patients included those diagnosed with an STS outside of the hair-bearing scalp or benign histologies (n=14). Chart review was conducted to extract data on demographics, tumor, and treatment characteristics for our final study population (n=95). LR was defined as recurrence at or near the location of the primary tumor after a period with no clinically detectable disease. All cases were reviewed by a second investigator to ensure accurate data acquisition. All ambiguities in data abstraction were reviewed and consensus was developed by at least two investigators.

Statistical analyses were performed using SAS software, version 9.4 (SAS Institute Inc., Cary, USA). The primary outcomes analysed were LRFS and, for patients with LR, LR after salvage therapy. Secondary outcomes analysed were distant relapse-free survival (DRFS) and OS. Kaplan-Meier statistics were used to estimate LRFS, DRFS, and OS. The date of histopathologic diagnosis was used to calculate outcomes. Cases were censored based on the date of last follow-up. The log-rank test was used to compare survival curves. Regression analyses to examine predictors of LR were calculated using the Cox proportional hazards model. Statistical significance was established at a p-value <0.05.

## Results

The clinical and treatment characteristics of the entire cohort and according to margin status are summarized in Table 1. Median follow-up time was 33 months (range 1.5-256 months). The median age at diagnosis was 77 years (range 19-96 years), and 80% were males. The most common histologies were angiosarcoma (27%), UPS (24%), and PDS (21%). Tumor grade was high in 43 patients, low in 4, and unknown in 48. Initial surgery was excisional biopsy in 60% of patients (n=57). Initial margin status was positive in 78% (n=74), close in 15% (n=14), negative in 5% (n=5), and missing in 2% (n=2). Positive initial margins were more likely in patients who underwent excisional biopsy (p=0.004) and those with angiosarcoma (p=0.049). Re-excision was more commonly performed after a positive initial margin (p=0.036) and was completed in 57% of patients (n=54). During re-excisions, 52% required a skin graft (n=28) and 30% required a tissue flap (n=16). Final margins were positive in 36% of patients (n=34), close in 28% (n=27), negative in 31% (n=29) and missing in 5% (n=5).

**Table 1:** Clinical and Treatment Characteristics of Entire Cohort and According to Initial Margin Status. *Statistically significant value, p <0.05.

			Initial margin status			
		All patients	Positive	Close (≤3 mm)	Negative	p-value
n, (%)		95 (100)	74 (78)	14 (15)	5 (5)	
Median follow-up, months (range)		33.1 (1.5-256)	33.1 (1.5-256)	20.7 (1.6-225)	70.8 (25-235)	0.145
Median age, months (range)		77 (19-96)	78 (19-96)	72 (20-88)	73 (56-88)	0.696
Sex, n (%)	Male	76 (80)	18 (24)	1 (7)	0	0.233
	Female	19 (20)	56 (76)	13 (93)	5 (100)	
Histology, n (%)	Angiosarcoma	26 (27)	25 (34)	1 (7)	0	0.049*
	Other	69 (73)	49 (66)	13 (93)	5 (100)	
Excisional biopsy as initial surgery, n (%)	Yes	57 (60)	38 (51)	13 (93)	4 (80)	0.004*
	No	38 (40)	36 (49)	1 (7)	1 (20)	
Re-excision extent, n (%)	Tissue flap	16 (17)	12 (16)	3 (21)	1 (20)	0.036*
	Skin graft	28 (30)	27 (36)	1 (7)	0	
	Other	10 (11)	9 (12)	0	1 (20)	
	None	41 (43)	26 (35)	10 (71)	3 (60)	
Closest margin location, n (%)	Radial	2 (2)	2 (3)	0	0	0.361
	Deep	15 (16)	15 (20)	0	0	
	Both	4 (4)	4 (5)	0	0	
	Not reported	74 (78)	53 (72)	14 (100)	5 (100)	
Re-excision included fascia, n (%)	Yes	15 (16)	14 (19)	1 (7)	0	0.645
	No	5 (5)	5 (7)	0	0	
	Not reported	75 (79)	55 (74)	13 (93)	5 (100)	
Radiotherapy, n (%)	Pre-operative	4 (4)	4 (5)	0	0	0.099
	Post-operative	26 (27)	25 (34)	1 (7)	0	
	None	65 (68)	45 (61)	13 (93)	5 (100)	

**Figure 1:**
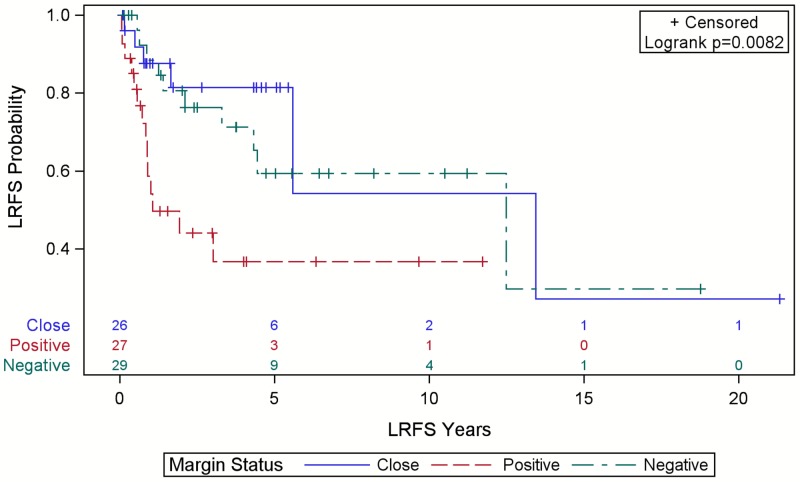
Local relapse-free survival (LRFS) for patients with scalp sarcoma by final margin status with numbers at risk Close margins were defined as ≤3 mm.

**Figure 2:**
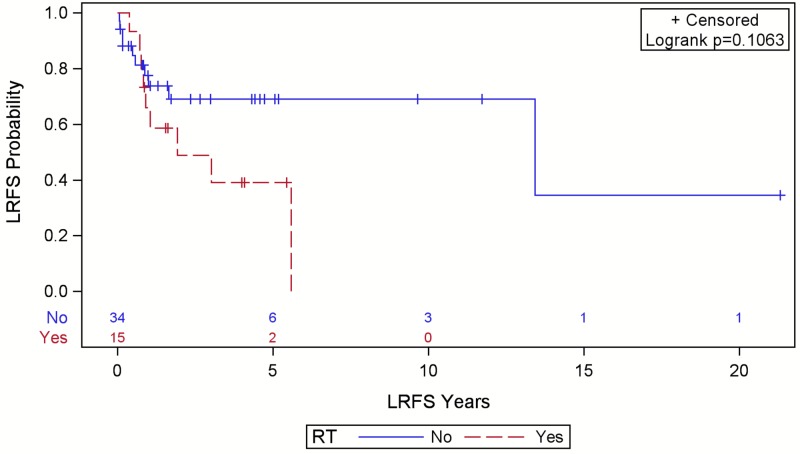
Local relapse-free survival (LRFS) in patients with close or positive final margins after surgery for scalp sarcoma by radiotherapy (RT) status with numbers at risk. Patients who received palliative-intent RT were excluded. Close margins were defined as ≤3 mm.

Local and distant recurrence outcomes are shown in Table 2. There were 32 LR (counting initial recurrences only), 18 DR, 21 sarcoma-specific deaths, and 53 all-cause deaths. Median survival was 54 months (95% confidence interval (CI): 31.8-84.2, range 1.5-256 months). Five-year OS was 48% (95% CI: 36.9-58.8). Median time to LR was 150 months (95% CI: 39.5 - not estimable) with a range of 0.8-256 months. Positive final margins were associated with worse LRFS (p=0.008) (Figure 1). Figure 2 shows no significant effect from RT on LRFS in patients with positive or close margins (p=0.106). Those who received palliative-intent RT were excluded (n=10). Pre-operative RT doses ranged from 50-60 Gray (Gy) in 25-30 fractions (median 53.5 Gy in 25 fractions) while post-operative RT doses ranged from 45-66 Gy in 15-33 fractions (median 60 Gy in 30 fractions). Radiation modalities included electrons only (n=9), photons only (n=6), a combination (n=5), or unknown (n=1).

**Table 2:** Local and Distant Recurrence Outcomes. LR = local recurrence, DR = distant recurrence, RT = radiotherapy.

	All patients		LR	DR
n (%)	95 (100)		32 (34)	18 (19)
Final margin status, n (%)				
Positive	34 (36)		14 (44)	13 (72)
Close (≤3 mm)	27 (28)		6 (19)	4 (22)
Negative	29 (31)		10 (31)	1 (6)
Not reported	5 (5)		2 (6)	0
Initial histology, n (%)				
Angiosarcoma	26 (27)		15 (47)	13 (72)
Undifferentiated pleomorphic sarcoma	23 (24)		5 (16)	3 (17)
Pleomorphic dermal sarcoma	20 (21)		6 (19)	2 (11)
Atypical fibroxanthomas	10 (11)		4 (13)	0
Dermatofibrosarcoma protuberans	10 (11)		1 (3)	0
Leiomyosarcoma	3 (3)		0	0
Solitary fibrous tumor	1 (1)		1 (3)	0
Myxofibrosarcoma	1 (1)		0	0
Fibrosarcoma	1 (1)		0	0
First LR characteristics				
LR treatment intent, n (%)				
Curative		16 (50)		
Palliative		16 (50)		
LR curative-intent treatment type, n (%)				
Surgery only		9 (56)		
Surgery + pre/post-operative RT		4 (25)		
Surgery + pre/post-operative RT + chemotherapy		2 (13)		
RT only		1 (6)		
DR characteristics				
Site of DR, n (%)				
Lung		6 (33)		
Neck lymph nodes		6 (33)		
Liver		2 (11)		
Skin		2 (11)		
Bone		1 (6)		
Not reported		1 (6)		
DR detection method, n (%)				
History and physical exam		10 (56)		
Other imaging		5 (28)		
Chest x-ray		3 (17)		

Half of the 32 first LR were treated with curative-intent/salvage treatment. Of these, nine had surgery only, four had surgery plus pre- or post-operative RT, two had tri-modality (surgery, RT, and chemotherapy), and one had RT only. Of the nine patients who had surgery only, three had been treated with RT as part of their initial treatment. All six cases that had a second LR were among those treated with surgery only after their first LR. Only one of these six cases had had prior RT and could not receive RT as part of their salvage treatment plan. The site of metastasis for the 18 patients that had DR were the neck lymph nodes (n=6), lung (n=6), liver (n=2), skin (n=2), bone (n=1), and not reported (n=1).

**Figure 3:**
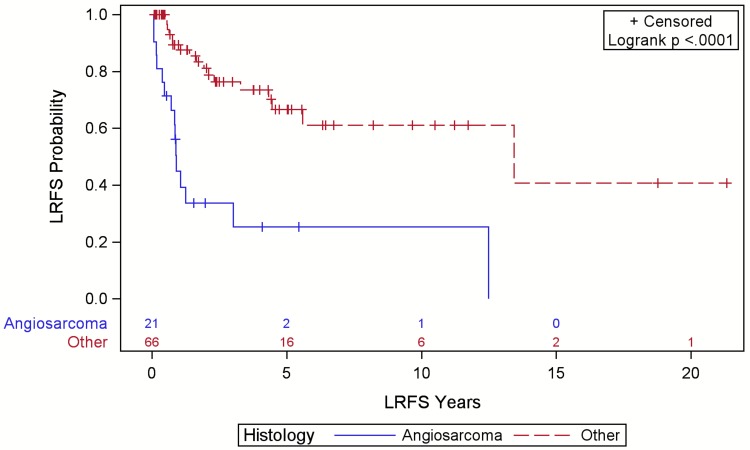
Local relapse-free survival (LRFS) for patients with scalp sarcoma by histology with numbers at risk.

**Figure 4:**
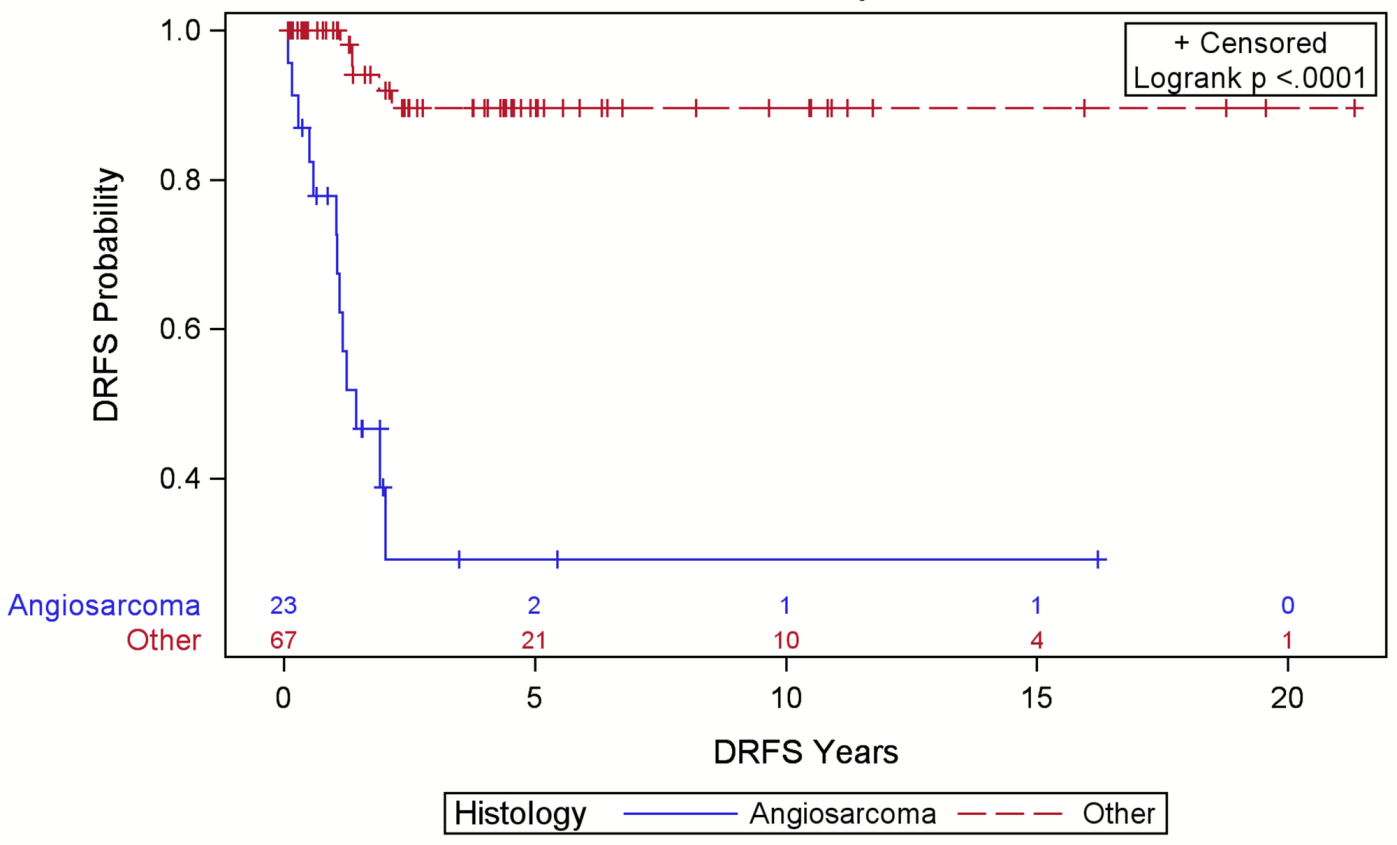
Distant relapse free survival (DRFS) for patients with scalp sarcoma by histology with numbers at risk.

**Figure 5:**
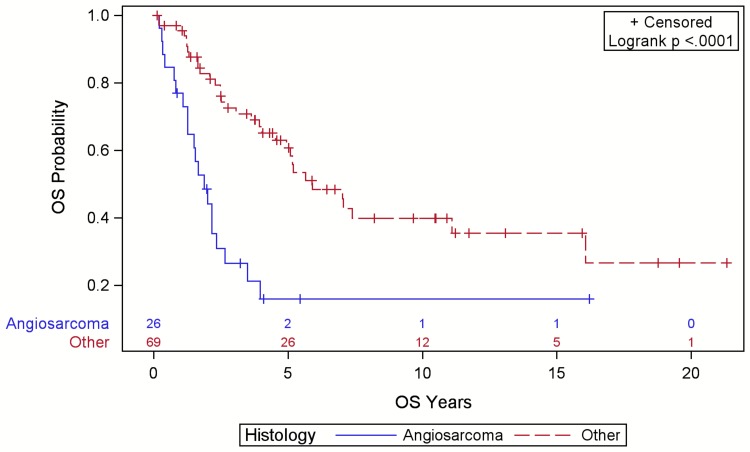
Overall survival (OS) for patients with scalp sarcoma by histology with numbers at risk.

Angiosarcoma was associated with reduced LRFS (Figure 3), DRFS (Figure 4), and OS (Figure 5) compared to other sarcoma histologies (p<0.001). The five-year LRFS was 10%, DRFS was 9%, and OS was 8% for angiosarcoma cases. Of 32 LR, 15 (47%) were angiosarcomas, six (19%) were PDS, five (16%) were UPS, four (13%) were AF, one (3%) was DFPS, and one (3%) was a solitary fibrous tumor. Of the 18 DR, 13 (75%) were angiosarcomas, three (17%) were UPS, and two (11%) were PDS. The majority of these DR were detected on history and physical exam (56%).

On univariate and multivariate analyses (Table 3) age, sex, final margin status, pre- or post-operative RT, and re-excision were not associated with a higher LR risk (all p>0.05). Pre- or post-operative RT approached, but did not reach, statistical significance on multivariate analysis with a hazard ratio (HR) of 0.37 and p-value of 0.066 (95% CI: 0.13-1.07). Both univariate and multivariate analyses show an increased risk of LR for angiosarcoma histologies (HR 4.81, p<0.001; HR 12.0, p<0.001; respectively).

**Table 3:** Regression analyses of factors associated with local recurrence HR : hazard ratio; CI : confidence interval; RT : radiotherapy.

	Univariate			Multivariate		
Variable	HR	95% CI	p-value	HR	95% CI	p-value
Age	1.02	1.00 – 1.05	0.084	1.01	0.98 – 1.04	0.368
Sex						
Female	Referent			Referent		
Male	1.76	0.62 – 5.04	0.290	1.92	0.60 – 6.21	0.274
Final margin status						
Negative	Referent			Referent		
Close/Positive	1.48	0.69 – 3.18	0.314	1.69	0.74 – 3.85	0.216
RT						
No RT	Referent			Referent		
Pre/Post-operative RT	1.80	0.87 – 3.72	0.110	0.37	0.13 – 1.07	0.066
Histology						
Other	Referent			Referent		
Angiosarcoma	4.81	2.34 – 9.87	<0.001*	12.06	4.10 – 35.5	<0.001*

## Discussion

Our results highlight the many challenges of treating scalp STS. Surgery and radiotherapy can be challenging, and LR rates are high compared to other STS sites. OS is poor, and many patients are still passing away from their disease.

Margin Status

The majority of patients initially underwent an excisional biopsy which was associated with a higher likelihood of positive initial margins and is a suboptimal initial surgical approach given STS often traverse their pseudocapsule and track along tissue planes [10]. Prior excision makes it harder to achieve wide surgical margins given fascial planes have been disrupted, tumor spread can be facilitated by wound hematomas, and it can be difficult to delineate both the residual tumor as well as the tissue that may have been contaminated [10]. We observed that re-excisions for scalp STS often required more extensive surgical procedures to preserve the functionality and cosmesis of the defect. Just over half of patients with close or positive initial margins underwent further surgery. De Bree et al. reviewed nine studies looking at a total of 996 patients and found that negative margins were obtained in 46% of head and neck STS versus 66% of STS located elsewhere [11]. Our 30% negative final margin rate further emphasizes the complexity of achieving a wide local excision on the scalp. De Bree et al. also found final margin status to be a statistically significant prognostic factor in six of eight studies who reported LR rates [11]. This correlates with the worse LRFS we observed in our population with positive final margins.

Adjuvant Radiotherapy and Local Relapse

Historically adjuvant RT for STS has been associated with improved LC [12]. This is evident in the meta-analysis of 3958 patients by Albertsmeier et al. which showed that RT reduced LR for non-retroperitoneal STS but did not improve OS [12]. Tran et al. reviewed 165 head and neck STS cases and found a significant benefit with adjuvant RT versus surgery alone on recurrence-free survival (87% vs 45%) [13]. They also showed a LC benefit to adjuvant RT for 27 patients with positive final margins (75% vs 26%) [13]. Barker et al. similarly found a LC benefit to adjuvant RT in a population of 44 patients with head and neck STS (54% vs 25%) [14].

Our analysis, however, found no statistically significant benefit to adjuvant RT for close or positive final margins in scalp STS. Additionally, a trend of lower LRFS was observed in those with close or positive margins who received RT, suggesting that those who were offered pre- or post-operative RT may have had higher risk disease than those who were not. In an attempt to adequately power our study to detect a difference in LRFS, we included intermediate risk histologies and low grade disease in our study population. Pre- or post-operative RT may be used less frequently in these groups, and this may have positively contributed to the LFRS in the population that did not receive RT. Even within high risk histologies, angiosarcomas are consistently shown to be highly malignant with high LR and DR rates and often receive RT as part of their treatment plan [15-17]. Our population contained a high percentage of angiosarcomas as compared to other STS sites [15]. Thus, it is likely that our RT cohort included more malignant histologies and higher grade disease.

Conversely, on multivariable analysis investigating the effect of radiotherapy on LC within our entire cohort, the HR approached but did not reach statistical significance. This suggests that the addition of radiotherapy to any treatment plan, regardless of final margin status, may decrease LR risk, but this cannot be concluded from our data alone. The low number of events observed within our population makes definitive interpretation of a multivariable analysis challenging.

Overall Survival

Galy-Bernadoy and Garrel reviewed 16 studies on head and neck STS with a total 1591 patients and found a mean five-year OS of 60% [18]. Similarly a five-year OS of 61% was found by Mahmoud et al. in a cohort of 788 high-grade head and neck STS patients [19]. Our five-year OS of 48% suggests a worse prognosis for patients diagnosed with scalp STS [19]. An older median age as well as an increased incidence of angiosarcomas within our population likely contributes to this [20]. Other factors associated with worse OS include complex anatomy which influences surgical resectability, as previously discussed, and radiation feasibility. The scalp is a challenging location to radiate due to field size and the curvature of the head with potential for severe toxicities including tissue necrosis when using an adequate pre- or post-operative sarcoma dose [15]. With the advent of intensity-modulated radiation therapy (IMRT) and volume-modulate radiation therapy (VMAT) more homogeneous dose distributions with reduced dose and volume to organs at risk is promising but not yet published for an analogous population [15].

Angiosarcoma

The large proportion of angiosarcomas within our cohort prompted further investigation of their outcomes compared to other histologies. The current standard of care remains wide surgical excision with adjuvant RT and consideration of chemotherapy [15,17,21]. Shin et al. completed a meta-analysis investigating 11 studies which included 379 patients treated for angiosarcomas of the scalp or face. The mean five-year OS was 33.5% while age ≥70, tumor size ≥5 cm, and scalp location were all associated with worse five-year OS. Final margin status was not shown to affect five-year OS. They found surgery was the most effective treatment for increasing five-year OS compared to RT and chemotherapy. For 24 patients who received curative-intent treatment for angiosarcomas of the scalp, Bernstein et al. reported a five-year locoregional control rate of 9%, five-year recurrence-free survival of 5%, and five-year OS of 9% [16]. These rates are similar to what we observed in our population regardless of treatment intent (five-year LRFS of 10%, five-year DRFS of 9%, and five-year OS of 8%) highlighting the very poor prognosis of scalp angiosarcomas.

The National Comprehensive Cancer Network (NCCN) reports the most common site of metastasis for extremity, superficial trunk, and head and neck STS is the lung [7]. We observed similar cases of lung metastasis and neck lymph node metastasis in our cohort. Angiosarcomas again proved to be more malignant in behavior as 13 of 18 DR were angiosarcomas. The higher proportion of angiosarcoma histologies in our population likely increased the risk of lymphatic DR compared to other STS sites [18].

Limitations

Our study is limited by the small sample size and retrospective design. While a multi-institutional prospective study with adequate power is needed, feasibility to accrue large patient samples is limited given the rarity of this disease. Thus, we rely on retrospective studies which have inherent bias. For example, patients with more significant clinical disease were most likely to undergo multimodality treatment leading to selection bias within our population. Additionally, as our study spanned 25 years, there were heterogeneities of treatment techniques. Advances in technology resulted in the evolution of RT treatment modalities including IMRT and VMAT which can create more complex dose distributions with more conformal, homogeneous plans. This could theoretically improve LR, but this has not yet been demonstrated. Few patients were treated alike limiting the generalizability of our results and possibly diluting any LR benefit from modern modalities.

## Conclusions

The rarity of scalp STS hinders the ability to formulate evidence-based guidelines for their treatment. They possess a higher risk of LR especially in cases with positive margins or angiosarcoma histology. Adjuvant RT was not associated with improved LC for close or positive margins, but our study was likely underpowered to detect an association. Further investigation would require a multi-institutional study. Until then complete surgical excision to establish negative margins remains the primary treatment modality.
